# SEDENTARY BEHAVIOR AND PHYSICAL ACTIVITY IN THE ADULT CHANGES IN THOUGHT (ACT) STUDY

**DOI:** 10.1093/geroni/igz038.071

**Published:** 2019-11-08

**Authors:** Dori E Rosenberg, Andrea Z LaCroix, Jack Guralnik

**Affiliations:** 1 Kaiser Permanente Washington Health Research Institute, Seattle, Washington, United States; 2 University of California San Diego, La Jolla, California, United States; 3 University of Maryland School of Medicine, Baltimore, Maryland, United States

## Abstract

Few epidemiologic studies have examined device-measured sitting patterns and health outcomes. Furthermore, there is a need to continue understanding the role of prospectively measured physical activity in relation to older adult health. The Adult Changes in Thought (ACT) study is an on-going epidemiologic study of adults age ≥65 years that began in 1994. Participants complete biennial assessments including a self-reported measure of physical activity. Starting in 2016, ACT participants could enroll in a physical activity sub-study that involved wearing a thigh-worn activPAL device and maintaining sleep logs for 7 days. Of those approached to participate in the sub-study, 64% agreed (N = 1139). A total of 961 had valid wear time (≥4 days with 10-20 hours of data per day) and completed survey collecting measures on pain and built environments (56% female, 57% > age 75, 89% non-Hispanic white). Participants who consented to the sub-study were generally younger and had fewer chronic conditions than those who did not consent. After removing sleep time, mean daily activPAL measures calculated included hours sitting and standing, number of sitting bouts lasting 30 minutes or more, number of breaks from sitting, and steps walked. The first session in this symposium will present historical self-reported physical activity trajectories in relation to cognitive function. The subsequent sessions will present novel cross-sectional data examining activPAL variables with measures of physical function, pain, and perceived built environments. This symposium will provide new insights on the roles of sedentary behavior and physical activity in aging and health.

